# Quality of relationships as predictors of outcomes in people with dementia: a systematic review protocol

**DOI:** 10.1136/bmjopen-2015-010835

**Published:** 2016-04-04

**Authors:** Hannah B Edwards, Jelena Savović, Penny Whiting, Verity Leach, Alison Richards, Sarah Cullum, Richard Cheston

**Affiliations:** 1 School of Social and Community Medicine, University of Bristol and National Institute for Health Research (NIHR) Collaboration for Leadership in Applied Health Research and Care (CLAHRC) West, Bristol, UK; 2 School of Social and Community Medicine, University of Bristol, Bristol, UK; 3 University of the West of England, Bristol, UK

**Keywords:** Family relationships, Carers, Institutionalisation, Challenging behaviour, Systematic review

## Abstract

**Introduction:**

Serious adverse outcomes for people with dementia include institutionalisation, hospitalisation, death, development of behavioural and psychiatric symptoms, and reduced quality of life. The quality of the relationship between the person with dementia and their informal/family carer is thought to affect the risk of these outcomes. However, little is known about which aspects of relationship quality are important, or how they affect outcomes for people with dementia.

**Methods and analysis:**

This will be a systematic review of the literature. Electronic databases MEDLINE, EMBASE, Web of Science, PsycInfo, the Cochrane Database, ALOIS and OpenGrey will be searched from inception. 2 independent reviewers will screen results for eligibility with standardised criteria. Data will be extracted for relevant studies, and information on the associations between relationship quality and dementia outcomes will be synthesised. Meta-analysis will be performed if possible to calculate pooled effect sizes. Narrative synthesis will be performed if study heterogeneity rules out meta-analysis.

**Ethics and dissemination:**

Ethical review is not necessary as this review summarises data from previous studies. Results will be disseminated via peer-reviewed publication. Results will also be disseminated to a patient and public involvement group and an expert panel for their views on the findings and implications for future work.

**Trial registration number:**

CRD42015020518.

**Strengths and limitations of this study**

- This systematic review will provide a comprehensive evaluation of how the quality of the relationship between patients with dementia and their informal carers influences adverse outcomes for patients.
- Results may help to inform future work to support families affected by dementia, with the aim of reducing or delaying institutionalisation.
- There may be significant heterogeneity in study methodology which could limit the strength of the conclusions we can draw from this review.

## Introduction

The WHO and International Classification of Diseases V.10 (ICD-10) define dementia as “a syndrome due to disease of the brain, usually of a chronic or progressive nature, in which there is disturbance of multiple higher cortical functions, including memory, thinking, orientation, comprehension, calculation, learning capacity, language, and judgement. Consciousness is not clouded. The impairments of cognitive function are commonly accompanied, and occasionally preceded, by deterioration in emotional control, social behaviour, or motivation. This syndrome occurs in Alzheimer disease, in cerebrovascular disease, and in other conditions primarily or secondarily affecting the brain.”^1^

Dementia is considered a major public health problem and is a recognised priority for the UK and other governments. It is a leading cause of disability and dependency in older people^2^ with high social and economic costs from medical and health and social care, informal care needs, and lost productivity. It is estimated to be the most expensive of any mental health or brain disorder in the UK,^3^ more expensive than heart disease, stroke or cancer.^4^ It accounts for 1% of total worldwide gross domestic product (GDP; up to 1.24% of total GDP in high-income countries).^5^ Worldwide, between 5% and 7% of people over 60 have dementia (more than 35 million people in 2010) with numbers predicted to double every 20 years.^6–8^ In the UK, in 2015, there are thought to be over 856 700 people with dementia, affecting 1 in every 14 people over age 65.^7^

Currently, little is known about options for prevention, and medical treatment only reduces symptoms for a relatively small proportion of the overall population. Interventions focus largely on treatment and management of symptoms as they arise, in order to minimise poor outcomes. Institutionalisation (being placed in a care home) is generally considered a ‘poor’ or last-ditch outcome to be pursued only when independent living and informal care are no longer sustainable. Most people with dementia wish to live in their own home for as long as possible.^9^ Indeed, continuing to do so has been found to support autonomy, sense of identity, well-being and quality of life (QoL).^10^ Care home fees are also a serious financial burden for those living with dementia and their families.^11^ ^12^ There are also indirect costs to the public as fees are often supplemented via taxation. Unfortunately, it is not always possible for the person with dementia to continue to live at home, and a prime factor leading to the breakdown of care is that their caregiver is no longer able to sustain the demands of care giving.^13^ Thus, both in the UK and elsewhere, government policy has emphasised the need to provide effective support to families in order to help them cope in the expectation that this would delay, and in some cases, prevent, institutionalisation. At the same time, recent media attention on poor standards of care, negligence and abuse in some care homes has generated serious concerns about placing loved ones, or being placed oneself in a care home.^14–16^

There is a growing evidence base linking relationship factors (such as amount of interaction and intimacy, and coping strategies) to outcomes both for people with dementia and for their carers. The quality of the patient–carer relationship has been associated with the likelihood of institutionalisation as well as the rate of decline, stress levels and QoL of people with dementia.^17–26^ Reviews of interventions suggest that optimising aspects of the patient–carer relationship could potentially improve outcomes in dementia,^27–30^ but these are likely to be most effective if we can identify and target those families at high risk of poor outcomes.

This review will evaluate the evidence on how the quality of the relationship between a person with dementia and their carer affects the patient's risk of institutionalisation, hospitalisation, development of behavioural and psychiatric symptoms of dementia (BPSD/challenging behaviour), QoL, and death.

## Methods and analysis

A somewhat flexible approach will be necessary for this review, as it is difficult to know in advance the nature of the studies and data that may be available. We will clearly document and justify any decisions made regarding amendments to the inclusion/exclusion criteria and synthesis plan proposed in this protocol, should they become necessary. The details below reflect the planned methods at the outset of the review.

### Registration

This review is registered with the PROSPERO International Prospective Register of Systematic Reviews,^31^ registration number CRD42015020518. The protocol is reported in line with recommendations of the PRISMA-P statement (Preferred Reporting Items for Systematic Review and Meta-Analysis Protocols).^32^ Publication will be reported in line with the PRISMA statement.^33^ Protocol amendments will be updated on PROSPERO.

### Inclusion criteria

*Study type*: We plan to include cohort studies as the most robust methodology for the research question. However, at the screening stage of the review, we will also record case–control and cross-sectional studies. If the synthesis of cohort studies alone does not yield a sufficient body of evidence to draw any useful conclusions, we will at that stage cautiously consider including case–control and cross-sectional studies. Intervention studies would be included if they specifically report data on the associations of exposures and outcomes for the control group (eg, a cohort study nested within a randomised controlled trial (RCT)). Relevant systematic reviews will be obtained and used as a means of identifying additional primary studies. Case reports, qualitative studies, cost-effectiveness studies, group-level/ecological studies and editorials will be excluded. Conference proceedings will be included if they contain sufficient data to assess inclusion and extract results.

*Population*: The population of interest is people with dementia and their informal/unpaid carer(s). Carer(s) may be a spouse, child, other family member, or non-family such as a friend or neighbour. Professional paid carers are excluded as their relationship with a patient with dementia is expected to be qualitatively different. All types of dementia will be included. Alzheimer's, vascular dementia, mixed dementia, frontotemporal dementia and dementia with Lewy bodies are anticipated to be the most common. Studies on patients with dementia in Parkinson's, Huntington's, Creutzfeldt-Jakob Disease (CJD), Wernicke-Korsakoff syndrome and other conditions will be included if it is explicit that the population studied also have dementia. Studies of people with mild cognitive impairment will be excluded. Studies involving mixed populations where only a subgroup is eligible will only be included if stratified subgroup results are available for the eligible subgroup. If subgroup-specific results are not available, a decision will be made via consultation with content and methodological expert collaborators as to the likely effect on results of inclusion of the non-relevant groups. Where the effect is judged to be minimal, the study may be included and contamination issues discussed. Case-by-case decisions will be added to the protocol to serve as a precedent for future cases.

*Exposures/risk factors studied*: Studies will be included if they measure an element of the quality of relationship between the person with dementia and their carer. Amount of contact, closeness, attachment, expressed emotion and coping style are primary exposures of interest. Measures of relationship quality prior to dementia onset and concurrently with dementia will both be included, although as far as possible, these will be analysed separately as relationships are expected to change as a result of dementia.^34^

Studies specifically focused on carer abuse, such as those that only include participants who are in abusive relationships (as defined by study authors), will be excluded. The rationale is that abuse is an ‘extreme’ dimension of relationship quality belonging to a different area of research (‘elder abuse’, which tends to focus on long-term abusive relationships and in the broader context of ageing, and does not focus specifically on dementia). It has also been dealt with elsewhere.^35^ ^36^

Studies in which participants in abusive relationships are included alongside participants in non-abusive relationships, which are also exploring our specified eligible exposures (and outcomes) of interest, will be included (eg, studies comparing risk of institutionalisation in participants in abusive relationships vs those in non-abusive relationships).

*Outcomes*: Outcomes of interest are for the person with dementia. The primary outcome is institutionalisation, that is, being placed in a care home/nursing home. Secondary outcomes are: death, acute or psychiatric hospitalisation, QoL, and development/progression of BPSD (also called ‘challenging behaviour’, which includes, eg, depression, anxiety, agitation, aggression and psychosis). Studies with BPSD/challenging behaviour or QoL as outcomes must use validated assessment tools.

There are no geographical or language restrictions on included studies. In cases where the full text is not available in English, attempts will be made to extract relevant data from the abstract (usually available in English) and results tables. Any cases where this is not possible will be reported. There are no date restrictions on inclusion; all databases will be searched from inception onwards.

### Search methods

A search strategy has been developed by an information scientist/research librarian (AR) in collaboration with content experts (RC and SC) who advised on relevant studies. Electronic databases MEDLINE, EMBASE, Web of Science, PsycInfo, The Cochrane Database and OpenGrey will be searched (see online supplementary material for the full search strategy). Bibliographies of included reports and relevant reviews will be screened to identify further potentially relevant reports. These will be included or excluded following the screening process described below.

10.1136/bmjopen-2015-010835.supp1 Supplementary data

### Selection of studies

*Selection of studies*: Title and abstract of all identified reports will be screened for relevance independently by two reviewers and all potentially relevant reports will be retrieved. Any discrepancies between the reviewers will be resolved via discussion, with other team members consulted where necessary. All retrieved papers will be read in full and assessed for eligibility against a standardised inclusion checklist applied by two reviewers independently. Reasons for exclusion will be recorded for all reports excluded at this stage. Discrepancies between the reviewers will be resolved via discussion, with other team members consulted where necessary.

### Data management and extraction

All reports identified in the search will be saved into Endnote X7 reference manager software, and after removal of duplicates, exported into a bespoke-built Microsoft Access database for management and screening. Data will be extracted onto a standardised electronic data collection form within the Access database. This form will be developed and piloted on five reports prior to use in the review. A second reviewer will check data extracted for errors and completeness. Checks will be escalated if there is a high error rate.

*The following information will be extracted*: bibliographic information, country of study, study design, sample size, participant characteristics (eg, age, gender, type of dementia, type of carer (eg, spouse, child, sibling)), exposures (eg, closeness, attachment, emotional expression) and outcomes (eg, institutionalisation, development of challenging behaviour, QoL) measured, measurement methods (eg, interview, survey, notes review, standardised psychological test), statistical methods, results, and authors' conclusions.

*Measures of effect/association*: For dichotomous data, data from 2×2 tables will be collected with associated risk ratios or ORs and 95% CIs. For continuous data, the mean difference between groups with 95% CIs will be collected. Both adjusted and unadjusted results will be collected where given.

### Data synthesis

Characteristics of included studies will be presented as a narrative summary or table, including study design, aims, population, setting, assessments and outcomes. If the data are too heterogeneous to pool, then narrative synthesis will be used to present results. Studies will be grouped by outcome, with descriptive text and tables used to summarise the range of results. For each outcome category, relationship factors would be grouped together. For example, for the outcome ‘institutionalisation’, we would present all results on the association of attachment style with the risk of institutionalisation, followed by the association of expressed emotion with the risk of institutionalisation, etc (The effect of these different exposures would not be combined in meta-analysis.) Commentary will detail how differences in methodologies used and potential biases could be affecting each study's results.

If there are a sufficient number of studies with sufficiently homogeneous populations, exposures and outcomes, data will be pooled and meta-analysis performed to calculate summary measures of effect. A random-effects model is likely to be most appropriate to allow for expected differences between studies.

The effect of relationship factors may plausibly vary by subgroups, for example, type of relationship (spouse vs parent–child vs other relative vs neighbour/friend), type/severity of dementia and the cultural setting. If there are sufficient studies with these data, we will perform subgroup analysis on these factors. If data pooling and subgroup analysis is not appropriate, where possible results for relevant subgroups will be reported and interpreted separately.

We will investigate whether the conclusions are robust to the exclusion of studies at high risk of bias. We plan to formulate main conclusions based on results from studies at low risk of bias.

### Assessment of methodological quality and risk of bias

Study quality and risk of bias will be assessed using the Newcastle-Ottawa scale.^37^ Part of this includes assessment of whether studies have adequately adjusted for confounding factors. Factors considered a priori potential confounders are age,^24^ gender,^38^ socioeconomic status,^39^ ethnicity,^40–42^ dementia type,^43^ dementia severity,^44^ BPSD,^45^ carer comorbidity,^46^ employment status of carer^47^ and alcohol consumption.^48^

*Assessment of heterogeneity*: Studies may be heterogeneous in a variety of ways including design factors, exposures studied, methods of assessment, participant factors (age, gender, ethnicity, carer type), setting (country, cultural group, community vs institution) and dementia type. This methodological and clinical heterogeneity will be assessed by reviewers during the data extraction and synthesis, and in reporting, we will comment on whether studies adjusted for potential baseline confounding factors.

If results allow, statistical heterogeneity will be explored by visual assessment of study results presented in a forest plot. Little overlap between CIs indicates heterogeneity. A χ^2^ statistic will be used to assess whether differences in results are compatible with chance alone, and I^2^ statistic to quantify the proportion of the total variability in results estimated to be due to study-specific heterogeneity, rather than random sampling error. Roughly, I^2^ of 0–40% can be interpreted as little heterogeneity, 30–60% as moderate, 50–90% substantial and 75–100% as considerable heterogeneity.^49^

*Assessment of reporting biases*: Historically, funnel plots have often been used as a method of assessing reporting bias. However, current expert consensus indicates caution in their use, as there are many possible alternative causes of funnel plot asymmetry, particularly in observational studies.^50^ The circumstances where interpretation of funnel plot results can be clearer (eg, meta-analyses of prospectively registered RCT results) do not apply to this review, so funnel plots will not be used here. The potential influence of reporting bias will be addressed in the Discussion section.

## Ethics and dissemination

Ethical review is not necessary as this review summarises data from previous studies. Results will be disseminated via peer-reviewed publication. Results will also be disseminated to a patient and public involvement group and an expert panel for their views on the findings and implications for future work.
