# Supplementary material for: Quality of relationships as predictors of outcomes in people with dementia: a systematic review protocol
Source: BMJ Open. 2016 Apr 4;6(4):e010835. doi: 10.1136/bmjopen-2015-010835 (PMC4823463; doi:10.1136/bmjopen-2015-010835)
Supplement: Supplementary data [file bmjopen-2015-010835supp.pdf]

# SEARCH STRATEGY

**Systematic Review:** Individual and family relationships as predictors of outcomes in people affected by dementia: a systematic review

**Databases:** Embase, Medline, Psychinfo, Web of Science, Cochrane.
The search strategy for each database is detailed below.

## EMBASE

Database: Embase <1974 to 2015 May 11>
Search Strategy:
--------------------------------------------------------------------------------
1 *dementia/ or *alzheimer disease/ or *cadasil/ or *diffuse lewy body disease/ or *diffuse neurofibrillary tangles with calcification/ or exp *frontotemporal dementia/ or *hiv associated dementia/ or *multiinfarct dementia/ or *pick presenile dementia/ or *presenile dementia/ or exp *senile dementia/ (127188)
2 alzheimer$.ti,ab. (127578)
3 (dementia or demented).ti. (44409)
4 ((dementia or demented) adj4 (senile or senility or elder$ or person$ or subject$ or adult$ or patient$ or people)).ab. (32436)
5 or/1-4 (183931)
6 *caregiver/ or *caregiver burden/ or *caregiver support/ or caregiver strain index/ (15454)
7 *family relation/ or *family conflict/ (2945)
8 *child parent relation/ (18108)
9 emotional intelligence/ (1316)
10 emotional attachment/ (4306)
11 family coping/ or family functioning/ or family interaction/ (3562)
12 ((carer$ or caregiver$ or care-giver$ or pre-caregiv$ or caregiving or care-giving) adj4 (quality of life or Qol or conflict$ or morbidity or capabilit$ or depression or depressive or anxiety or closeness or motivat$ or resilien$ or communicat$ or support or counsel$ or informal or factor$ or distress$ or relationship$ or stress or burden$ or support$ or interaction$ or commitment or wellbeing or well-being or style$ or dysfunctional or dependen$ or independen$ or intimat$ or affection or affectionate or tension$ or pressure$ or disagreement$ or isolat$ or loving)).ti,ab. (20332)
13 ((marital or spous$ or couple$ or daughter$ or son or son$2 or partner$ or husband$ or wife$ or wives or familial or families) adj4 (quality of life or Qol or morbidity or counsel$ or support or depression or depressive or anxiety or closeness or motivat$ or resilien$ or capabilit$ or communicat$ or informal or factor$ or distress$ or relationship$ or stress or burden$ or support$ or interaction$ or commitment or wellbeing or well-being or conflict$ or style$ or dysfunctional or dependen$ or independen$ or intimat$ or affection or affectionate or tension$ or pressure$ or disagreement$ or isolat$ or loving)).ti,ab. (74168)
14 (Relationship$ adj3 (loving or intimate or intimacy or affection or affectionate or quality or close$ or dysfunctional or dependen$ or predictor$)).ti,ab. (36940)
15 new york university caregiver intervention.ti,ab. (7)
16 *marriage/ or *spouse/ (15122)
17 "sense of coherence"/ (641)
18 (expressed adj2 emotion).ti,ab. (1222)
19 ((role captivity or self efficacy or sense of competence or sense of coherence) and (carer$ or caregiver$ or care-giver$ or pre-caregiv$ or caregiving or care-giving or marital or spous$ or couple$ or daughter$ or son or son$2 or partner$ or husband$ or wife$ or wives or familial or families)).ti,ab. (3054)
20 (attachment adj2 (style$ or avoidant or insecure or secure or ambivalent)).ti,ab. (2432)

21 exp *adaptive behavior/ or *coping behavior/ (30848)
22 (coping adj2 (acceptance-based or solution-based or positive or abilit$ or strateg$ or style$ or mechanism$ or strain or stress)).ti,ab. (21085)
23 *social isolation/ or *loneliness/ (6328)
24 (social adj2 isolation).ti,ab. (5882)
25 (emotional adj2 (strain or impact or health or support)).ti,ab. (10379)
26 *social support/ or *respite care/ (15162)
27 ((loneliness or social support or time to listen or befriend$ or lines of communication) and (carer$ or caregiver$ or care-giver$ or pre-caregiv$ or caregiving or care-giving or marital or spous$ or couple$ or daughter$ or son or son$2 or partner$ or husband$ or wife$ or wives or familial or families)).ti,ab. (8387)
28 (respite adj2 (break$ or care)).ti,ab. (749)
29 or/6-28 (243201)
30 5 and 29 (7756)
31 letter.pt. or letter/ (889069)
32 note.pt. (598597)
33 editorial.pt. (476664)
34 case report/ or case study/ (2029980)
35 (letter or comment$).ti. (157341)
36 or/31-35 (3836762)
37 exp clinical study/ or meta analysis/ or "meta analysis (topic)"/ or "systematic review"/ or "systematic review (topic)"/ or cross-sectional study/ or crossover procedure/ or (systematic review or meta-analys$ or study or trial or cross-over or crossover or cross-sectional or case-control or cohort$ or blind$ or random$).mp. (14600734)
38 36 not 37 (1524603)
39 exp animal/ not human/ (4494915)
40 nonhuman/ (4504835)
41 exp experimental animal/ (457411)
42 exp animal experiment/ (1848987)
43 exp animal model/ (829666)
44 exp rodent/ (3024267)
45 (rat or rats or mouse or mice).ti. (1317756)
46 or/38-45 (8447146)
47 30 not 46 (7357)
48 meta analysis/ or "meta analysis (topic)"/ or "systematic review"/ or "systematic review (topic)"/ (166636)
49 (meta analy$ or metaanaly$ or metanaly$ or meta regression).ti,ab. (99458)
50 ((systematic$ or evidence$) adj2 (review$ or overview$)).ti,ab. (108142)
51 (reference list$ or bibliograph$ or hand search$ or manual search$ or relevant journals).ab. (33172)
52 (search strategy or search criteria or systematic search or study selection or data extraction).ab. (34138)
53 (search$ adj4 literature).ab. (40229)
54 (medline or pubmed or cochrane or embase or psychlit or psyclit or psychinfo or cinahl or science citation index or bids or cancerlit).ab. (123876)
55 cochrane.jw. (12418)
56 ((multiple treatment$ or indirect or mixed) adj2 comparison).ti,ab. (1799)
57 or/48-56 (317594)
58 randomized controlled trial/ or "randomized controlled trial (topic)"/ (442912)
59 crossover procedure/ (42753)
60 "double blind procedure"/ (122609)
61 "single-blind procedure"/ (20152)
62 (randomi$ or randomly).ti,ab. (795220)
63 trial.ti. (181805)
64 (cross-over$ or crossover$ or cross over$).ti,ab. (75959)

65 ((doubl$ or singl$) adj blind$).ti,ab. (171033)
66 or/58-65 (1067625)
67 Clinical study/ (110797)
68 exp case control study/ (99829)
69 family study/ (10770)
70 longitudinal study/ (76925)
71 retrospective study/ (399400)
72 prospective study/ (289051)
73 cross-sectional study/ (143173)
74 cohort analysis/ (200367)
75 follow up/ (910549)
76 cohort$.ti,ab. (471173)
77 75 and 76 (98758)
78 (cross-sectional or case-control or interrupted time series).ti,ab. (337093)
79 (cohort adj (study or studies or analys$)).ti,ab. (138586)
80 ((follow up or followup or observational or non randomi#ed or uncontrolledl or "before and after") adj2 (study or studies)).ti,ab. (156520)
81 ((longitudinal$ or retrospectiv$ or prospectiv$) adj6 (study or studies or review or analys$ or cohort$)).ti,ab. (884195)
82 or/67-74,77-81 (1856248)
83 57 or 66 or 82 (2929368)
84 prognosis/ (453898)
85 disease course/ (317345)
86 treatment outcome/ or outcome assessment/ (929562)
87 disease severity assessment/ or apache/ (9250)
88 kaplan meier method/ (38059)
89 patient acuity/ (279)
90 "severity of illness index"/ (8484)
91 statistical model/ (115081)
92 exp regression analysis/ or statistical analysis/ (469010)
93 proportional hazards model/ or multivariate analysis/ (156362)
94 (likelihood or probabilit$ or predict$ or prognos$ or progress$ or logistic regression).ti,ab. (2886071)
95 ((multivariate or regression) adj2 analys$).ti,ab. (389260)
96 ((cox or linear or hazard$ or mixed or proportional or logistic) adj3 model$).ti,ab. (182048)
97 institutionalization/ or institutionali$.ti,ab. (18870)
98 or/84-97 (4319535)
99 83 or 98 (6105919)
100 47 and 99 (3985)

## MEDLINE

Database: Medline In-process - Current week, Medline 1950 to present
Search Strategy:
--------------------------------------------------------------------------------
1 dementia/ or aids dementia complex/ or alzheimer disease/ or exp aphasia, primary progressive/ or exp dementia, vascular/ or diffuse neurofibrillary tangles with calcification/ or exp frontotemporal lobar degeneration/ or lewy body disease/ (111181)
2 alzheimer$.ti,ab. (98468)
3 (dementia or demented).ti. (33717)
4 ((dementia or demented) adj4 (person$ or senile or senility or elder$ or subject$ or adult$ or patient$ or people)).ab. (23494)
5 or/1-4 (151599)

6 *Caregivers/ or caregivers/px (18460)
7 family relations/ or family conflict/ (9371)
8 intergenerational relations/ or parent-child relations/ (31073)
9 emotional intelligence/ (1004)
10 *interpersonal relations/ (23404)
11 family/px or spouses/ or marriage/px (26847)
12 ((carer$ or caregiver$ or care-giver$ or pre-caregiv$ or caregiving or care-giving) adj4 (quality of life or Qol or conflict$ or morbidity or capabilit$ or depression or depressive or anxiety or closeness or motivat$ or resilien$ or communicat$ or support or counsel$ or informal or factor$ or distress$ or relationship$ or stress or burden$ or support$ or interaction$ or commitment or wellbeing or well-being or style$ or dysfunctional or dependen$ or independen$ or intimat$ or affection or affectionate or tension$ or pressure$ or disagreement$ or isolat$ or loving)).ti,ab. (14943)
13 ((marital or spous$ or couple$ or daughter$ or son or son$2 or partner$ or husband$ or wife$ or wives or familial or families) adj4 (quality of life or Qol or morbidity or counsel$ or support or depression or depressive or anxiety or closeness or motivat$ or resilien$ or capabilit$ or communicat$ or informal or factor$ or distress$ or relationship$ or stress or burden$ or support$ or interaction$ or commitment or wellbeing or well-being or conflict$ or style$ or dysfunctional or dependen$ or independen$ or intimat$ or affection or affectionate or tension$ or pressure$ or disagreement$ or isolat$ or loving)).ti,ab. (60894)
14 (Relationship$ adj3 (loving or intimate or intimacy or affection or affectionate or quality or close$ or dysfunctional or dependen$ or predictor$)).ti,ab. (30669)
15 new york university caregiver intervention.ti,ab. (5)
16 Expressed Emotion/ (1466)
17 sense of coherence/ (315)
18 (expressed adj2 emotion).ti,ab. (997)
19 ((role captivity or self efficacy or sense of competence or sense of coherence) and (carer$ or caregiver$ or care-giver$ or pre-caregiv$ or caregiving or care-giving or marital or spous$ or couple$ or daughter$ or son or son$2 or partner$ or husband$ or wife$ or wives or familial or families)).ti,ab. (2199)
20 (attachment adj2 (style$ or avoidant or insecure or secure or ambivalent)).ti,ab. (1866)
21 *adaptation, psychological/ (33853)
22 (coping adj2 (acceptance-based or solution-based or positive or abilit$ or strateg$ or style$ or mechanism$ or strain or stress)).ti,ab. (16036)
23 social isolation/ or loneliness/ (13056)
24 (social adj2 isolation).ti,ab. (4417)
25 (emotional adj2 (strain or impact or health or support)).ti,ab. (7768)
26 *social support/ or respite care/ (19752)
27 ((loneliness or social support or time to listen or befriend$ or lines of communication) and (carer$ or caregiver$ or care-giver$ or pre-caregiv$ or caregiving or care-giving or marital or spous$ or couple$ or daughter$ or son or son$2 or partner$ or husband$ or wife$ or wives or familial or families)).ti,ab. (6261)
28 (respite adj2 (break$ or care)).ti,ab. (591)
29 or/6-28 (255631)
30 5 and 29 (7435)
31 letter/ (878639)
32 editorial/ (377659)
33 news/ (168499)
34 exp historical article/ (333775)
35 Anecdotes as topic/ (4623)
36 comment/ (626697)
37 case report/ (1731222)
38 (letter or comment).ti. (66298)
39 or/31-38 (3462608)

40 exp clinical trial/ or exp clinical trials as Topic/ or meta-analysis/ or meta-analysis as topic/ or cross-over studies/ or exp epidemiologic studies/ or (systematic review or meta-analys$ or study or trial or cross over or crossover or cross sectional or case control or cohort$ or blind$ or random$).mp. (7668336)
41 39 not 40 (3093302)
42 animals/ not humans/ (3943260)
43 exp Animals, Laboratory/ (745617)
44 exp Animal Experimentation/ (6628)
45 exp Models, Animal/ (436913)
46 exp rodentia/ (2736988)
47 (rat or rats or mouse or mice).ti. (1142420)
48 or/41-47 (7676181)
49 30 not 48 (6825)
50 meta-analysis/ (55637)
51 meta-analysis as topic/ (14222)
52 (meta analy$ or metaanaly$ or metanaly$ or meta regression).ti,ab. (77420)
53 ((systematic$ or evidence$) adj2 (review$ or overview$)).ti,ab. (88839)
54 (reference list$ or bibliograph$ or hand search$ or manual search$ or relevant journals).ab. (28082)
55 (search strategy or search criteria or systematic search or study selection or data extraction).ab. (30051)
56 (search$ adj4 literature).ab. (32198)
57 (medline or pubmed or cochrane or embase or psychlit or psyclit or psychinfo or cinahl or science citation index or bids or cancerlit).ab. (100580)
58 cochrane.jw. (11498)
59 ((multiple treatment$ or indirect or mixed) adj2 comparison).ti,ab. (1034)
60 or/50-59 (227071)
61 randomized controlled trial.pt. or randomized controlled trial/ or Randomized Controlled Trials as Topic/ (486988)
62 controlled clinical trial.pt. (89427)
63 (randomi$ or randomly).ti,ab. (604805)
64 trial.ti. (138376)
65 clinical trials as topic.sh. (172816)
66 or/61-65 (1005304)
67 epidemiologic studies/ (6184)
68 cross-over studies/ (35964)
69 exp case control studies/ (713979)
70 exp cohort studies/ (1432164)
71 cross-sectional studies/ (193001)
72 "Controlled Before-After Studies"/ (35)
73 interrupted time series analysis/ (35)
74 case control.ti,ab. (85271)
75 (cohort adj (study or studies or analys$)).ti,ab. (104741)
76 ((follow up or followup or uncontrolled or non randomi#ed or observational or "before and after") adj2 (study or studies)).ti,ab. (116321)
77 ((longitudinal$ or retrospectiv$ or prospectiv$) adj6 (study or studies or review or analys$ or cohort$)).ti,ab. (627937)
78 cross sectional.ti,ab. (192858)
79 interrupted time series.ti,ab. (1226)
80 (control$ adj4 (group$ or trial or study)).ti,ab. (593653)
81 (cross-over$ or crossover$ or cross over$).ti,ab. (65294)
82 or/67-81 (2506236)
83 prognosis/ or survival analysis/ or kaplan-meier estimate/ or treatment outcome/ or "Outcome Assessment (Health Care)"/ (1117694)
84 Disease Progression/ (110573)

85 health status indicators/ or apache/ or patient acuity/ or "severity of illness index"/ (201441)
86 (likelihood or probabilit$).ti,ab. (223333)
87 (predict$ or prognos$ or progress$).ti,ab. (2002491)
88 models, statistical/ or likelihood functions/ or linear models/ or logistic models/ or proportional hazards models/ or regression analysis/ or least-squares analysis/ (389820)
89 ((multivariate or regression) adj2 analys$).ti,ab. (287877)
90 logistic regression.ti,ab. (152336)
91 ((cox or linear or hazard$ or mixed or proportional or logistic) adj3 model$).ti,ab. (131889)
92 Institutionalization/ or Nursing Homes/ut or institutionali$.ti,ab. (15705)
93 or/83-92 (3413126)
94 60 or 66 or 82 or 93 (5399217)
95 49 and 94 (3874)

## PSYCHINFO

Database: PsycINFO <1806 to May Week 1 2015>
Search Strategy:
--------------------------------------------------------------------------------
1 dementia/ or aids dementia complex/ or dementia with lewy bodies/ or presenile dementia/ or semantic dementia/ or exp senile dementia/ or vascular dementia/ or alzheimer's disease/ or picks disease/ (56057)
2 alzheimer$.ti,ab. (42977)
3 (dementia or demented).ti. (22125)
4 ((dementia or demented) adj4 (elder$ or senile or senility or person$ or subject$ or adult$ or patient$ or people)).ti,ab. (18797)
5 or/1-4 (66161)
6 ((carer$ or caregiver$ or care-giver$ or pre-caregiv$ or caregiving or care-giving) adj4 (quality of life or Qol or conflict$ or morbidity or capabilit$ or depression or depressive or anxiety or closeness or motivat$ or resilien$ or communicat$ or support or counsel$ or informal or factor$ or distress$ or relationship$ or stress or burden$ or support$ or interaction$ or commitment or wellbeing or well-being or style$ or dysfunctional or dependen$ or independen$ or intimat$ or affection or affectionate or tension$ or pressure$ or disagreement$ or isolat$ or loving)).ti,ab. (15209)
7 ((marital or spous$ or couple$ or daughter$ or son or son$2 or partner$ or husband$ or wife$ or wives or familial or families) adj4 (quality of life or Qol or morbidity or counsel$ or support or depression or depressive or anxiety or closeness or motivat$ or resilien$ or capabilit$ or communicat$ or informal or factor$ or distress$ or relationship$ or stress or burden$ or support$ or interaction$ or commitment or wellbeing or well-being or conflict$ or style$ or dysfunctional or dependen$ or independen$ or intimat$ or affection or affectionate or tension$ or pressure$ or disagreement$ or isolat$ or loving)).ti,ab. (63974)
8 (Relationship$ adj3 (loving or intimate or intimacy or affection or affectionate or quality or close$ or dysfunctional or dependen$ or predictor$)).ti,ab. (25220)
9 new york university caregiver intervention.ti,ab. (3)
10 (expressed adj2 emotion).ti,ab. (1585)
11 ((role captivity or self efficacy or sense of competence or sense of coherence) and (carer$ or caregiver$ or care-giver$ or pre-caregiv$ or caregiving or care-marital or spous$ or couple$ or daughter$ or son or son$2 or partner$ or husband$ or wife$ or wives or familial or families)).ti,ab. (2851)
12 (attachment adj2 (style$ or avoidant or insecure or secure or ambivalent)).ti,ab. (6161)
13 (coping adj2 (acceptance-based or solution-based or positive or abilit$ or strateg$ or style$ or mechanism$ or strain or stress)).ti,ab. (28270)
14 (social adj2 isolation).ti,ab. (5075)
15 (emotional adj2 (strain or impact or health or support)).ti,ab. (9683)

16 ((loneliness or social support or time to listen or befriend$ or lines of communication) and (carer$ or caregiver$ or care-giver$ or pre-caregiv$ or caregiving or care-marital or spous$ or couple$ or daughter$ or son or son$2 or partner$ or husband$ or wife$ or wives or familial or families)).ti,ab. (8486)
17 (respite adj2 (break$ or care)).ti,ab. (560)
18 caregivers/ or caregiver burden/ or elder care/ (24619)
19 *family relations/ (24979)
20 *interpersonal relationships/ or exp family conflict/ (14094)
21 intergenerational relations/ (3128)
22 parent child relations/ (23963)
23 emotional intelligence/ (3760)
24 *couples/ or *spouses/ (13434)
25 *marital status/ or *marriage/ (7397)
26 expressed emotion/ (1291)
27 "Sense of Coherence"/ (1168)
28 *coping behavior/ (31605)
29 social isolation/ (5821)
30 loneliness/ (3182)
31 *Social Support/ (20990)
32 respite care/ (401)
33 or/6-32 (249997)
34 5 and 33 (7103)
35 (authored book or book or edited book).pt. (409358)
36 scientific communication/ (21960)
37 case report/ (22551)
38 (letter or comment$).ti. (33887)
39 35 or 36 or 37 or 38 (482275)
40 (random$ or trial or blind$ or prognos$ or prospectiv$ or retrospectiv$ or "before and after" or interrupted time series or case control or cross-sectional or crossover or cross-over or cohort$ or meta-analys$ or systematic review or longitudinal).ti,ab. (464856)
41 39 not 40 (459334)
42 exp animals/ or animal models/ (290922)
43 (rat or rats or mouse or mice).ti. (96299)
44 or/41-43 (745544)
45 34 not 44 (6512)
46 meta analysis/ (3649)
47 (meta analy$ or metaanaly$ or metanaly$ or meta regression).ti,ab. (20935)
48 ((systematic$ or evidence$) adj2 (review$ or overview$)).ti,ab. (23038)
49 (reference list$ or bibliograph$ or hand search$ or manual search$ or relevant journals).ab. (17934)
50 (search strategy or search criteria or systematic search or study selection or data extraction).ab. (3271)
51 (search$ adj4 literature).ab. (6117)
52 (medline or pubmed or cochrane or embase or psychlit or psyclit or psychinfo or cinahl or science citation index or bids or cancerlit).ab. (13402)
53 cochrane.jw. (0)
54 ((multiple treatment$ or indirect or mixed) adj2 comparison).ti,ab. (124)
55 clinical trials/ (8640)
56 randomi#ed.ab. (48449)
57 random$.ti,ab. (141165)
58 placebo.ab. (31763)
59 randomly.ab. (54448)
60 trial.ti. (19386)
61 case control.ti,ab. (7274)
62 (cohort adj (study or studies or analys$)).ti,ab. (12120)

63 ((follow up or followup or observational or "before and after" or uncontrolled or non randomi#ed) adj (study or studies)).ti,ab. (17233)
64 ((longitudinal$ or prospectiv$) and (study or studies or studied or review$ or analys$ or cohort$)).ti,ab. (104849)
65 cross sectional.ti,ab. (45320)
66 interrupted time series.ti,ab. (494)
67 followup studies/ (12328)
68 cohort analysis/ (1118)
69 exp longitudinal studies/ (15563)
70 (crossover$ or cross-over$).ti,ab. (7590)
71 or/46-70 (394874)
72 prediction/ (17035)
73 prognosis/ (7200)
74 exp multivariate analysis/ (16733)
75 exp statistical regression/ (4806)
76 Statistical Analysis/ (14428)
77 statistical probability/ (4016)
78 item response theory/ (4659)
79 least squares/ (680)
80 treatment outcomes/ (26880)
81 disease course/ (9928)
82 "severity (disorders)"/ (13060)
83 (likelihood or probabilit$ or predict$ or prognos$ or progress$).ti,ab. (496092)
84 ((multivariate or regression) adj2 analys$).ti,ab. (71311)
85 logistic regression.ti,ab. (31604)
86 ((cox or linear or hazard$ or mixed or proportional or logistic) adj3 model$).ti,ab. (28503)
87 institutionalization/ (3346)
88 institutionali$.ti,ab. (13041)
89 or/72-88 (630258)
90 71 or 89 (906642)
91 45 and 90 (2901)

## WEB OF SCIENCE

| | | |
|---|---|---|
| # 31 | 3,896 | #30 AND #19<br>*Indexes=SCI-EXPANDED, SSCI, CPCI-S, CPCI-SSH Timespan=All years* |
| # 30 | 6,282,730 | #29 OR #28 OR #27 OR #26 OR #25 OR #24 OR #23 OR #22 OR #21 OR #20<br>*Indexes=SCI-EXPANDED, SSCI, CPCI-S, CPCI-SSH Timespan=All years* |
| # 29 | 266,576 | TS= ((cox or linear or hazard* or mixed or proportional or logistic) near/3 model*)<br>*Indexes=SCI-EXPANDED, SSCI, CPCI-S, CPCI-SSH Timespan=All years* |
| # 28 | 336,935 | TS= ((multivariate or regression) near/2 analys*)<br>*Indexes=SCI-EXPANDED, SSCI, CPCI-S, CPCI-SSH Timespan=All years* |
| # 27 | 3,829,258 | TS= (likelihood or probabilit* or predict* or prognos* or progress* or "logistic regression" or institutionali* )<br>*Indexes=SCI-EXPANDED, SSCI, CPCI-S, CPCI-SSH Timespan=All years* |
| # 26 | 839,359 | TS= (control* near/4 (group* or trial or study))<br>*Indexes=SCI-EXPANDED, SSCI, CPCI-S, CPCI-SSH Timespan=All years* |
| # 25 | 384,811 | TS= (case-control or cross-sectional or "interrupted time series" or crossover* or cross-over* )<br>*Indexes=SCI-EXPANDED, SSCI, CPCI-S, CPCI-SSH Timespan=All years* |

| | | |
|---|---|---|
| # 24 | 710,670 | TS= ((cohort near/2 stud*) or (cohort near/2 analys*) or ("before and after" near/2 stud*) or (longitudinal* near/6 stud*) or (longitudinal* near/6 analys*) or (follow-up near/2 stud*) or (followup near/2 stud*) or (observational near/2 stud*) or (retrospectiv* near/6 stud*) or (retrospectiv* near/6 analys*) or (prospectiv* near/6 stud*) or (prospectiv* near/6 analys*) or (longitudinal* near/6 cohort*) or (retrospectiv* near/6 cohort*) or (prospectiv* near/6 cohort*))<br>*Indexes=SCI-EXPANDED, SSCI, CPCI-S, CPCI-SSH Timespan=All years* |
| # 23 | 264,330 | TI= trial*<br>*Indexes=SCI-EXPANDED, SSCI, CPCI-S, CPCI-SSH Timespan=All years* |
| # 22 | 1,242,539 | TS= random*<br>*Indexes=SCI-EXPANDED, SSCI, CPCI-S, CPCI-SSH Timespan=All years* |
| # 21 | 204,404 | TS= ((controlled near/2 "clinical trial") or (doubl* near/2 blind*) or (singl* near/2 blind*) or (trebl* near/2 blind*) or (tripl* near/2 blind*))<br>*Indexes=SCI-EXPANDED, SSCI, CPCI-S, CPCI-SSH Timespan=All years* |
| # 20 | 287,295 | TS= (metaanalys* or meta-analys* or "meta regression" or metanalys* or (systematic near/2 review*) or (systematic near/2 overview*) or (evidence* near/2 review*) or (evidence* near/2 overview*) or (search* near/4 literature*) or (hand near/2 search*) or (manual* near/2 search*) or (search near/2 strateg*) or (systematic* near/2 search*) or "study selection" or "data extraction" or "search criteria" or (indirect near/2 comparison) or (mixed near/2 comparison) or ("multiple treatment" near/2 comparison) or ("multiple treatments" near/2 comparison))<br>*Indexes=SCI-EXPANDED, SSCI, CPCI-S, CPCI-SSH Timespan=All years* |
| # 19 | 7,212 | #15 not #18<br>*Indexes=SCI-EXPANDED, SSCI, CPCI-S, CPCI-SSH Timespan=All years* |
| # 18 | 2,175,105 | #17 OR #16<br>*Indexes=SCI-EXPANDED, SSCI, CPCI-S, CPCI-SSH Timespan=All years* |
| # 17 | 198,475 | TS= ((animal* near/2 experiment*) or (animal* near/2 model*) or (animal* near/2 laborator*))<br>*Indexes=SCI-EXPANDED, SSCI, CPCI-S, CPCI-SSH Timespan=All years* |
| # 16 | 2,020,675 | TI= (rat or rats or mouse or mice or rodent* or comment* or letter or "case study" or "case report" or anecdote* or editorial* or news )<br>*Indexes=SCI-EXPANDED, SSCI, CPCI-S, CPCI-SSH Timespan=All years* |
| # 15 | 7,434 | #14 AND #3<br>*Indexes=SCI-EXPANDED, SSCI, CPCI-S, CPCI-SSH Timespan=All years* |
| # 14 | 324,066 | #13 OR #12 OR #11 OR #10 OR #9 OR #8 OR #7 OR #6 OR #5 OR #4<br>*Indexes=SCI-EXPANDED, SSCI, CPCI-S, CPCI-SSH Timespan=All years* |
| # 13 | 222,937 | TS= ((carer* or caregiver* or care-giver* or pre-caregiv* or caregiving or care-giving or marital or spous* or couple* or daughter* or son or sons or partner* or husband* or wife* or wives* or family or families or familial) near/4 ("quality of life" or qol or conflict* or morbidity or capabilit* or depression or depressive or anxiety or closeness or motivat* or resilien* or communicat* or support* or counsel* or informal or factor* or distress* or relationship* or stress or burden* or interaction* or commitment or wellbeing or well-being or style* or dysfunctional or dependen* or independen* or intimat* or intimacy or affection* or tension* or pressure* or disagreement* or isolat* or loving))<br>*Indexes=SCI-EXPANDED, SSCI, CPCI-S, CPCI-SSH Timespan=All years* |
| # 12 | 57,045 | TS= (relationship* near/3 (loving or intimat* or intimacy or affection* or quality or close* or dysfunctional or dependen* or predictor*))<br>*Indexes=SCI-EXPANDED, SSCI, CPCI-S, CPCI-SSH Timespan=All years* |
| # 11 | 4,080 | TS= (attachment near/2 (style* or avoidant or insecure or secure or ambivalent)) |

| | | |
|---|---|---|
| | | *Indexes=SCI-EXPANDED, SSCI, CPCI-S, CPCI-SSH Timespan=All years* |
| # 10 | 29,472 | TS= (coping near/2 (acceptance-based or solution-based or positive or abilit* or strateg* or style* or mechanism* or strain or stress))<br>*Indexes=SCI-EXPANDED, SSCI, CPCI-S, CPCI-SSH Timespan=All years* |
| # 9 | 10,767 | TS= (emotional near/2 (strain or impact or health or support))<br>*Indexes=SCI-EXPANDED, SSCI, CPCI-S, CPCI-SSH Timespan=All years* |
| # 8 | 619 | TS= (respite near/2 (break or care))<br>*Indexes=SCI-EXPANDED, SSCI, CPCI-S, CPCI-SSH Timespan=All years* |
| # 7 | 5,581 | TS= (social near/2 isolation)<br>*Indexes=SCI-EXPANDED, SSCI, CPCI-S, CPCI-SSH Timespan=All years* |
| # 6 | 21,636 | TS= (("role captivity" or "self efficacy" or "sense of competence" or "sense of coherence" or loneliness or "social support" or "time to listen" or befriend* or "lines of communication" ) and (carer* or caregiver* or care-giver* or pre-caregiv* or caregiving or care-giving or marital or spous* or couple* or daughter* or son or sons or partner* or husband* or wife* or wives* or family or families or familial))<br>*Indexes=SCI-EXPANDED, SSCI, CPCI-S, CPCI-SSH Timespan=All years* |
| # 5 | 7 | TS= "new york university caregiver intervention"<br>*Indexes=SCI-EXPANDED, SSCI, CPCI-S, CPCI-SSH Timespan=All years* |
| # 4 | 4,134 | TS=(expressed near/2 emotion)<br>*Indexes=SCI-EXPANDED, SSCI, CPCI-S, CPCI-SSH Timespan=All years* |
| # 3 | 199,635 | #2 OR #1<br>*Indexes=SCI-EXPANDED, SSCI, CPCI-S, CPCI-SSH Timespan=All years* |
| # 2 | 193,800 | TI= (dementia or demented ) or TS= alzheimer*<br>*Indexes=SCI-EXPANDED, SSCI, CPCI-S, CPCI-SSH Timespan=All years* |
| # 1 | 36,758 | TS= ((dementia or demented) near/4 (elder* or senile or senility or person* or individual* or subject* or adult* or patient* or people*))<br>*Indexes=SCI-EXPANDED, SSCI, CPCI-S, CPCI-SSH Timespan=All years* |

## COCHRANE

Search Name: dementia_relation_prog
Date Run: 12/05/15 15:08:16.546
Description:

ID Search Hits
#1 MeSH descriptor: [Dementia] this term only 1443
#2 MeSH descriptor: [AIDS Dementia Complex] this term only 55
#3 MeSH descriptor: [Alzheimer Disease] this term only 2258
#4 MeSH descriptor: [Aphasia, Primary Progressive] explode all trees 8
#5 MeSH descriptor: [Dementia, Vascular] explode all trees 299
#6 MeSH descriptor: [Frontotemporal Lobar Degeneration] explode all trees 14
#7 MeSH descriptor: [Lewy Body Disease] this term only 48
#8 alzheimer*:ti,ab 5005
#9 (dementia or demented):ti 3405
#10 ((dementia or demented) near/3 (elder* or senile or senility or person* or subject* or adult* or patient* or people)):ti,ab 2209
#11 #1 or #2 or #3 or #4 or #5 or #6 or #7 or #8 or #9 or #10 8250
#12 MeSH descriptor: [Caregivers] this term only1281
#13 MeSH descriptor: [Family Relations] this term only 183
#14 MeSH descriptor: [Family Conflict] this term only 47

#15 MeSH descriptor: [Intergenerational Relations] this term only 32
#16 MeSH descriptor: [Parent-Child Relations] this term only 719
#17 MeSH descriptor: [Emotional Intelligence] this term only 31
#18 MeSH descriptor: [Interpersonal Relations] this term only 1584
#19 MeSH descriptor: [Spouses] this term only 223
#20 MeSH descriptor: [Marriage] this term only and with qualifier(s): [Psychology - PX] 98
#21 MeSH descriptor: [Family] this term only and with qualifier(s): [Psychology - PX] 404
#22 MeSH descriptor: [Expressed Emotion] this term only 122
#23 MeSH descriptor: [Sense of Coherence] this term only 7
#24 MeSH descriptor: [Adaptation, Psychological] this term only 3138
#25 MeSH descriptor: [Social Isolation] this term only 141
#26 MeSH descriptor: [Loneliness] this term only56
#27 MeSH descriptor: [Social Support] this term only 2467
#28 MeSH descriptor: [Respite Care] this term only 33
#29 (coping near/2 (acceptance-based or solution-based or positive or abilit* or strateg* or style* or mechanism* or strain or stress)):ti,ab 1119
#30 (social near/2 isolation):ti,ab 157
#31 ((loneliness or "social support" or "time to listen" or befriend* or "lines of communication") and (carer* or caregiver* or care-giver* or pre-caregiv* or caregiving or care-giving or marital or spous* or couple* or daughter* or son or sons or partner* or husband* or wife* or wives* or familial or family or families)):ti,ab 486
#32 (emotional near/2 (strain or impact or health or support)):ti,ab 475
#33 (respite near/2 (break* or care)):ti,ab 28
#34 "new york university caregiver intervention":ti,ab 2
#35 (relationship* near/3 (loving or intimate or intimacy or affection* or quality or close* or dysfunctional or dependen* or predictor*)):ti,ab 714
#36 (expressed near/2 emotion):ti,ab 107
#37 (("role captivity" or "self efficacy" or "sense of competence" or "sense of coherence") and (carer* or caregiver* or care-giver* or pre-caregiv* or caregiving or care-giving or marital or spous* or couple* or daughter* or son or sons or partner* or husband* or wife* or wives* or familial or family or families)):ti,ab 496
#38 (attachment near/2 (style* or avoidant or insecure or secure or ambivalent)):ti,ab 77
#39 ((carer* or caregiver* or care-giver* or pre-caregiv* or caregiving or care-giving or marital or spous* or couple* or daughter* or son or sons or partner* or husband* or wife* or wives* or familial or family or families) near/4 ("quality of life" or Qol or morbidity or conflict* or capabili* or depression or depressive or anxiety or anxieties or closeness or motivat* or resilien* or communicat* or support* or counsel* or informal or factor* or distress* or relationship* or stress or burden* or interaction* or commitment or wellbeing or well-being or style* or dysfunctional or dependen* or independen* or intimat* or intimacy or affection* or tension* or pressure* or disagreement* or isolat* or loving)):ti,ab 4968
#40 #12 or #13 or #14 or #15 or #16 or #17 or #18 or #19 or #20 or #21 or #22 or #23 or #24 or #25 or #26 or #27 or #28 or #29 or #30 or #31 or #32 or #33 or #34 or #35 or #36 or #37 or #38 or #39 14181
#41 #11 and #40 849
